# Iodine in Diphtheria

**Published:** 1881-03

**Authors:** 


Iodine in Diphtheria.—Dr. H. P. Gauthier, of St. Paul, Minn., reports astonishing results from the use of Iodine in Diphtheria. Thus during an epidemic of that disease in Natchez, Mississippi, in 1865, about a hundred cases were successfully treated with Iodine. He subsequently treated two hundred cases in Illinois, "all with the same satisfactory result, except in two instances where death occurred; the patient being almost moribund when coming under treatment." "The patient is ordered tincture of iodine in ten or twelve drop doses, every hour, well diluted with water, so long as the fever lasts, subsequently reducing it to ten drops every two, and finally, every three hours; local applications of the drug are made use of at the same time. These latter should be made by the physician himself, at least three times a day." He prefers the decolorized tincture for internal use and allows bread and starchy articles for food in abundance at the same time.

The foregoing is taken from the *Chicago Medical Review*, of Feb. 20th, ultimo. His success, as stated in the *Review*, is astounding; and we earnestly trust similar results may attend the use of the drug in other hands. If such should be the case the dreaded pestilence will be robbed of half its terrors. Will not some of our readers apply the remedy and send us reports of the results?

